# Enhancing Service Quality and Empowerment in Government Clinics Through Continuous Quality Improvement of Community Score Cards: A Case Study From the Dominican Republic

**DOI:** 10.9745/GHSP-D-24-00171

**Published:** 2026-08-10

**Authors:** Erin Morse, Mary Mukomba, Jose Eduardo Rodriguez, Kristen Mallory, Christian Taco, Cesar Jacome Castro, Enmanuel Díaz Santiago, Anne K. Sebert Kuhlmann

**Affiliations:** a Children International, Kansas City, MO, USA.; b Children International, Lusaka, Zambia.; c Children International, Santo Domingo, Dominican Republic.; d Children International, Quito, Ecuador.; e Children International, Barranquilla, Colombia.; f Saint Louis University, College of Public Health and Social Justice, St. Louis, MO, USA.

## Abstract

**Background:**

The Dominican Republic offers universal, government-run health care through primary, secondary, and tertiary levels. Reliability and confidence in care have lower user satisfaction than other dimensions. Thus, the government aims to enhance relationships between service users and providers as a key health systems strengthening priority to improve primary care services.

**Program Description:**

From 2018 to 2023, a global nonprofit organization implemented social accountability, using an adapted Community Score Card (CSC) process, in 10 government-run clinic sites in the Dominican Republic. Each site implemented multiple CSC cycles, which involved a facilitated dialogue process among service users, providers, and community leaders, prioritization, action planning, and implementation. We developed a comprehensive monitoring and evaluation toolkit to facilitate ongoing analysis and use of data for continuous quality improvement of the social accountability approach and sharing of the results with stakeholders. The toolkit includes components that span all steps in the CSC process, including tracking participation of key stakeholder groups, assessing CSC facilitation quality, scoring clinics on 4 defined global indicators (quality of care, availability of medical staff, availability of medicines, and community participation), tracking progress on action plans, tracking participants’ level of empowerment over time, and assessing how interventions impacted change through periodic use of the Most Significant Change monitoring and evaluation technique.

**Process Improvements:**

The data generated from the toolkit supported process improvements in the CSC approach, including the importance of youth leadership in social accountability and of achieving concrete change early on to drive the capacity for more complex change requiring vertical support. These iterative improvements to the CSC process resulted in empowerment and engagement of community members to drive change in government-run primary health clinics and improved perceptions of service quality. Case study data from the Dominican Republic show ongoing stakeholder participation, improvement across the 4 global indicators, and community empowerment, which collectively contribute to strengthening local health care services. The comprehensive toolkit supports efforts for continuous quality improvement while producing evidence locally, nationally, and globally for health systems strengthening and demonstrating the effectiveness of the CSC approach.

**Conclusions:**

Three key lessons emerged from the development of a CSC monitoring toolkit. First, a comprehensive toolkit centralizes data in one place and pulls together evidence from multiple sources. Second, a standardized toolkit allows for analysis at multiple levels. Finally, ensuring data are actionable locally is central to gathering complete, accurate data for continuous quality improvement.

## Abstract (precis)

A comprehensive Community Score Card monitoring and evaluation toolkit, integrating quantitative and qualitative data over multiple cycles of the intervention, helped drive continuous quality improvement in social accountability leading to the empowerment of health service providers, users, and youth to work together to improve government-run health clinics in the Dominican Republic.

## Key Messages

- While there is a growing body of evidence on the impact of social accountability approaches such as Community Score Cards on health care outcomes, gaps exist in monitoring and evaluating the processes undertaken with social accountability interventions.
- A comprehensive monitoring and evaluation toolkit for Community Score Cards provided local staff guidance and structure to collect and use relevant data to continuously improve social accountability program implementation.
- Meaningful youth engagement and leadership in social accountability, rather than mere participation, was found to be important for elevating issues important to youth.
- Community action to achieve discrete, concrete successes early during the Community Score Card process was also found to help empower stakeholders to advance more complex, vertical issues.

## INTRODUCTION

Social accountability has been increasingly recognized as a key element to improve the quality of health services.^1^^,^^2^ Guerzovich and Poli define social accountability as^3^:

*a process that enables the inclusive participation and collective action of citizens and civil society organizations in public policy making and implementation so that state and service providers are responsive to citizens’ needs and held accountable*.

A number of reviews have highlighted social accountability and its contributions toward improvement of service delivery, utilization, and health outcomes.^4^^–^^8^ In addition to health-related outcomes, governance outcomes, including participation, citizen empowerment, and health system responsiveness, have been associated with social accountability in some countries and contexts.^9^^–^^14^ Social accountability is considered a High Impact Practice for improving the delivery and utilization of family planning,^15^ a promising practice to improve respectful care,^16^ and a powerful platform for youth to hold health systems accountable.^17^

The Community Score Card (CSC), developed by CARE Malawi, is a collaborative and participatory social accountability approach that enables spaces for rights holders and duty bearers to assess the current situation of public services, develop joint action plans to address prioritized issues, and hold one another accountable.^13^^,^^18^ The CSC intervenes at the community level, which can lead to higher system actions and responsiveness through collective voice and advocacy of community members, civil society organizations (CSOs), and local-level duty bearers—also referred to as “vertical integration.”^19^ The CSC has proven effective in engaging community and health care providers to monitor and manage health facilities, particularly when faced with challenges of accountability, quality, and coverage in Bangladesh.^20^ Furthermore, a cluster randomized controlled trial in Malawi found that the CSC contributed to significant improvements in reproductive health-related outcomes.^13^

The CSC process integrates different methods of quantitative and qualitative data collection over multiple cycles, with the aim of Continuous Quality Improvement (CQI) and increased responsiveness. CQI has been utilized for health systems strengthening, enabling a “culture of continuous learning, innovation, and improvement” through a Plan, Do, Study/Check, and Act cycle to improve health service delivery.^21^ Core elements involve identifying problems, resolving problems, and encouraging a process of trust, respect, and empowerment while discouraging fear and blame.^21^^,^^22^ To enable CQI and effective use of data, it is critical to monitor and evaluate the CSC process. Monitoring and evaluating social accountability interventions is critical to support greater collective learning and strategic scale-up.^23^^–^^25^ Furthermore, there is growing evidence that supports the important role of youth in civic engagement and how to include them in social accountability, but there is need for additional evidence to understand the impact and most effective methods of youth engagement.^17^

Children International, a global nonprofit organization focused on ending generational poverty, is implementing social accountability using the CSC^13^ in 9 countries in Africa, Asia, and Latin America. While there is a growing body of evidence on social accountability approaches such as Community Score Cards to improve health care,^13^^,^^26^^–^^28^ there are gaps in monitoring and evaluation approaches that focus on the processes of social accountability interventions. In this article, we share a monitoring and evaluation system that we developed to support practitioners in the Dominican Republic to track their progress and continuously improve their CSC process while building evidence more effectively. The article includes results of how improvements in the health system were advanced but does not aim to provide direct evidence for health outcome improvements due to CSC.

## THE CSC PROCESS

Children International has operated for 45 years in the Dominican Republic in 4 provinces and 10 cities. In 2018, global staff introduced social accountability in the country through an adapted CSC process as an approach to improve local health care services. After training, local staff have been leaders and facilitators of the entire process.

The Dominican Republic offers universal health care through 3 distinct levels of service—primary, secondary, and tertiary care. The Ministry of Health is committed to improving quality of care throughout the system, especially at the primary care level, as established by the General Health Law 42-01.^29^ By law, primary care clinics are meant to serve approximately 500–700 families and provide the same package of primary care services for all ages, with typically a higher demand for services for children under 10 and adults over 35. In practice, the services offered vary by clinic and depend on factors such as the density of the community, the epidemiological profile of the community, demand of health services, and resources allocated to the clinic (economic and medical personnel). Given the priority for young and older populations, services tailored to the unique needs of adolescents is an identified gap in most clinic sites.^30^^,^^31^

Reliability and confidence in care are 2 dimensions the government has noted have lower levels of user satisfaction than other dimensions.^32^ Therefore, strengthening quality of care at government-run primary care clinics and improving relationships between service users and providers are system priorities. Building on the General Health Law 42-01, the government also established a guide for social participation in primary health care clinics, allowing the community to actively engage in decision-making, planning, execution, and evaluation of health policies and programs.^30^ This law established that all primary health clinics are required to have a committee that shares responsibility in ensuring that primary health care is more effective, equitable, and sustainable. The law and government commitment to social participation establishes an enabling environment for social accountability and for the CSC as a practical mechanism to support the government’s goals.

From 2018 to 2023, the Dominican Republic team expanded use of the CSC to 10 government-run clinic sites serving 21,700 people. The 10 clinics represent 8 diverse areas of the country–3 urban, 3 peri-urban, and 2 rural communities. In 2 sites (1 rural and 1 peri-urban), the clinics serve Haitian migrant communities, called Bateyes. A batey is a community built around sugar cane fields and production, with high levels of poverty and typically limited access to water, electricity, education, health care, employment, and legal counsel, particularly related to labor rights.^33^ Most batey residents are Haitian or Dominican-born children of Haitian migrants and lack legal status in the Dominican Republic.^34^ This creates unique challenges for the health clinic to provide adequate services. While the health authorities adapt clinic offerings based on community needs and demand, resources are inadequately distributed, leaving many clinics without services guaranteed by law and creating a need for people to seek services in secondary- and tertiary-level clinics.

Each of the 10 selected sites implemented between 2 to 7 cycles of the CSC, with each cycle lasting approximately 6 months. This process was led by a local facilitator assigned by Children International to each site. The facilitators played a crucial role in coordinating and guiding the activities in each cycle. In the Dominican Republic, as in all other places where Children International implements this process, facilitation is conducted in the country’s native language.

The program facilitator and the community center coordinator arranged and set up the discussion spaces where the meetings were held. Each cycle brought together service users (both adults and youth), health care providers, and community leaders, as community leaders are often users of the health care services themselves and are connected to the community’s reality.

**FIGURE 1:**
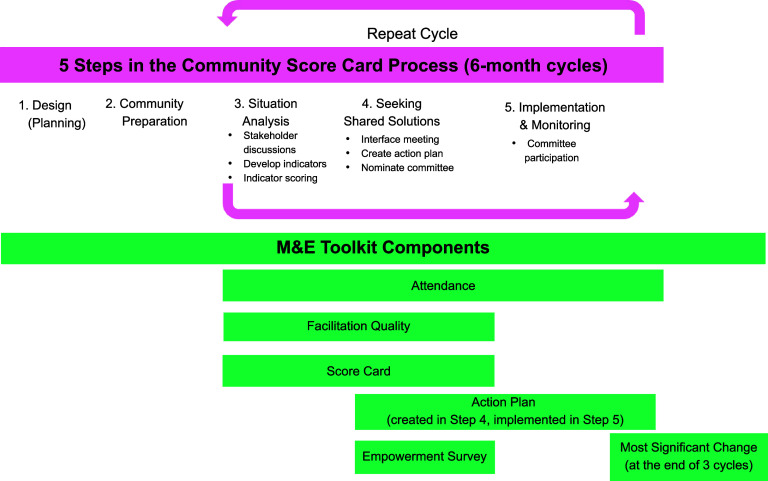
Steps of the Community Score Card Process with Associated Monitoring and Evaluation Toolkit Components

The CSC process used across the 10 sites consisted of 5 steps (Figure 1):

1. Design (Planning)
2. Community preparation
3. Situation analysis
4. Seeking shared solutions
5. Implementation and monitoring

Each step is outlined in Children International’s CSC Implementation Manual (Supplement 1), adapted from Care Malawi’s CSC manual.^18^ Step 1 includes staff planning, organizing and understanding the community through activities such as service mapping, transect walks, social mapping, and stakeholder analysis. Although staff members have established working relationships with community leaders, families, and youth, they take time to better understand the community through a new lens during this planning phase and cast a wide net to identify key stakeholders to invite to participate in the CSC process, by engaging with the health clinic staff, meeting with community leaders, reaching out to youth and families, and asking for recommendations. There is particular emphasis on identifying mothers who are often responsible for the health care of their family and vulnerable groups who may be uniquely impacted.

Step 2 focuses on orienting the community and generating buy-in from stakeholders, with a priority on youth, service users, providers, and community leaders.

During the first CSC cycle, the situation analysis in Step 3 includes focused discussion in separate groups of providers, service users, and community leaders to understand the situation. Staff and a representative group of stakeholders identify local indicators to track alongside the global indicators. Each stakeholder group scores the local and global indicators, and then the average score across the separate group scores is calculated. The stakeholder groups also select a solutions committee during Step 3, which makes the solutions committee a representative group of the entire community. In subsequent CSC cycles, the situation analysis includes an update from the solutions committee on progress made, reflection on the current situation, and then a rescoring of the indicators. Stakeholders recount their interactions with the health care services and share experiences from those indirectly involved in the CSC process. This process shares examples from across the entire community as there is an exchange of perspectives between youth, users, service providers, and community leaders.

Step 4, seeking solutions, brings stakeholders together through a facilitated dialogue to identify the reason for an indicator score, prioritize issues and build a vision for the future, and create objectives and an action plan to achieve them.

During the final step, implementation and monitoring, the solutions committee advances their action plan and checks in at least one time as a group after 3 months.

The entire CSC process relies on people to bring their personal perspectives and then come to a consensus through the group. Stakeholders frequently continue from cycle to cycle, providing continuity across cycles. When a new cycle starts every 6 months, however, new people may join, based on interest, referral, or transition of community leadership or clinic staff, creating a mix of new and continuing participants. Overall, across all sites in the Dominican Republic and all cycles of the CSC, there were 99 activities with a total of 1,771 participants, for an average of 17.8 participants per activity.

The CSC process is captured through a monitoring and evaluation toolkit designed to document evidence of change and to build a culture of continuous quality improvement within the program.

## MONITORING AND EVALUATION TOOLKIT

The effectiveness of social accountability approaches has been evaluated in a variety of ways with different methods.^23^^,^^24^ We believe that day-to-day monitoring and evaluation led by local staff to facilitate ongoing use of data for continuous improvement is critical to CSC success. At the same time, balancing the need to share data with local stakeholders while also meeting broader organizational needs for evidence of impact can be challenging. To meet these dual needs and enable CQI through the CSC, when we launched the social accountability approach in the Dominican Republic, it was also critical to embark on a participatory process to develop an accompanying CSC monitoring and evaluation system, including a comprehensive toolkit.

Throughout launching and expanding to new clinics, local, regional, and global staff worked together to develop and refine a monitoring and evaluation system to meet both local and organizational needs. The goal was to create easy-to-use tools that would provide local sites with data they could use, visualize, and share with key stakeholders, including local government officials, while also allowing headquarters to aggregate and summarize data across sites for their broader organizational evaluation needs. Draft tools were developed and a virtual training and feedback session was held with staff from multiple countries. The tools were revised and more specific guidance developed based on feedback from the local staff. Then, a second virtual training session in both English and Spanish by bilingual global staff was held. An additional in-person training was held in the Dominican Republic in Spanish, which provided feedback for further refinement of the tools.

The result was a comprehensive toolkit with 6 components—attendance, score card, facilitation quality, empowerment survey, action plan, and Most Significant Change—that is shared with each country and provided to each field staff working in CSC. The toolkit is a spreadsheet with different tabs for each component (Supplement 2). It is housed internally in shared folders so that each country and project site has access to their own files. Below, we describe each of the 6 components of the toolkit and how they relate to the 5 steps of CSC process (Figure 1).

### Attendance

Social accountability, as an approach, brings key stakeholders, including health services providers and users, especially vulnerable or marginalized service users who often have less power or voice in collective matters, together for facilitated dialogue. Interventions based on social accountability approaches often report the number of participants in various research or evaluation activities such as surveys or focus group discussions.^20^^,^^28^^,^^35^ Some report how many people participate in the actual social accountability process but may only report a range of participants at a specific phase^28^ or the number of facilities that participate. Very few report participation by type of stakeholder at various phases of the process. Our toolkit tracks attendance and participation by key stakeholder groups—providers, youth, community leaders, health services users—to ensure representation from vulnerable or marginalized groups, and attendance is tracked during all 5 steps of the CSC process. Attendance tracking also includes how many participants are repeat attendees of each session, an indicator of a higher level of engagement in the social accountability process than just participating once.

**FIGURE 2:**
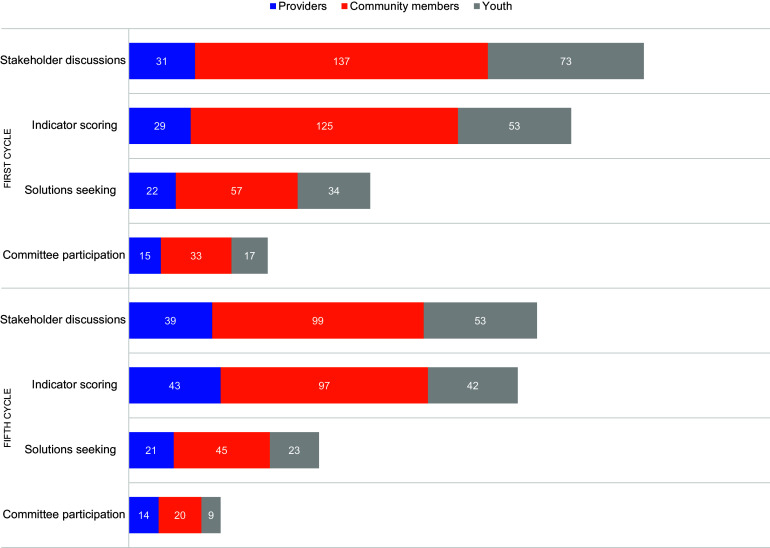
Number of CSC Participants by Stakeholder Group, First Cycle to Fifth Cycle, 9 Sites in the Dominican Republic, 2018 to 2023 Abbreviation: CSC, Community Score Card.

Figure 2 shows CSC participation by stakeholder across 9 sites in the Dominican Republic, from the first cycle to the fifth cycle, between 2018 and 2023. The data show that the sites have consistently engaged key stakeholder groups and kept them invested in participating over time. As expected, there is a slight decrease in participation from the initial launch, but after 3 years of ongoing work there is still consistent participation from providers, community members, and youth. The most important component of participation is the representation across different stakeholder groups in the community, especially groups that often have limited voice in community services, such as youth. While the absolute number of participants relative to the overall population of the catchment area is small, the involvement of a range of stakeholder groups, especially those whose voices are often marginalized in the community, is vitally important. Given the time commitment involved in the CSC process over a 6-month period, stakeholders such as youth participating both throughout the initial cycle and then continuing to participate from cycle to cycle shows the level of engagement that the process generates. In addition, the continued participation of stakeholders, such as youth, across 5 cycles of the CSC cycle over 3 years demonstrates how invested they are. Across all 10 sites in the Dominican Republic, 64.3% of participants in the second cycle of the CSC and beyond were continuing participants (data not shown). Many of the participants are repeat attendees from cycle to cycle; however, a few new individuals join during the situation analysis at the start of a new cycle, creating a mix of continuing and new participants.

At the local level, tracking attendance by stakeholder groups shows government officials that these stakeholders are engaged in the process, as intended. At the organizational level, we can aggregate attendance numbers across sites to highlight the level of community involvement that the intervention generates. Local staff in the Dominican Republic compare attendance data across sites, cycles, and steps of the process. The data were primarily managed by our staff. Due to a manual process for analysis, it was challenging to ensure all stakeholders were involved in reflecting on the data. An area of learning is to dedicate resources to conducting the analysis and presenting it in a way that is easily consumable and to exploring digital options to enable real-time data capture and analysis.

#### Youth Voice and Engagement

Through the attendance tracking data, we identified sites where youth were less involved and then generated tactics for increasing youth engagement through logistics and timing of meetings. We also pilot-tested a youth-led CSC process in 2 sites where youth participated not just as a stakeholder group but instead were part of the project design and were trained as co-facilitators. The overall focus of these sites was on making the clinics’ services more youth friendly. Through our experience with youth at these sites, the CSC process in the Dominican Republic has intentionally evolved to incorporate youth voice and perspective, with an ongoing commitment to enhancing youth engagement. In 3 of the 10 sites, youth were co-facilitators, supporting and leading facilitation of the entire CSC process. In these sites, more youth health issues were brought into the process and youth voice was more prominent in the process than the other 7 sites where youth participated as a stakeholder group. The involvement of youth in these 3 sites played a pivotal role in identifying unique health care needs specific to this demographic. For example, youth lobbied for improved youth-friendly spaces, improved sanitation facilities, and waiting chairs, which contributed to the ongoing improvement and responsiveness of the health care system. In addition, in youth-led sites, issues of access to sexual and reproductive health services emerged naturally.

### Facilitation Quality

Social accountability interventions designed to provide a safe space for dialogue and negotiation among diverse groups that often have differing power dynamics are predicated on strong, high-quality facilitation to elicit and manage this dialogue within the context of these power dynamics. Yet evaluations often overlook the role of facilitation quality in intervention effectiveness and rarely track facilitation quality.^22^ If interventions fail to produce the intended outcomes, it raises the question of whether poor-quality facilitation contributed to the lack of success or whether there was a larger theory failure in terms of the appropriateness of the intervention for the context.^36^

Prior to CSC implementation, our CSC Implementation Manual outlines facilitation guidance and desirable skills of a facilitator. Staff use the manual to identify experienced facilitators with strong facilitation skills and train them on the CSC process prior to launch. As new staff join, experienced staff train additional facilitators.

To assess facilitation quality, we developed a checklist based on 3 key dimensions^37^:

1. **Logistical**, which consists of 8 items that focus on preparedness for the session, starting on time, and following the agenda and procedures.
2. **Relational**, comprising 7 items that center around the facilitator’s interaction with participants, such as greeting attendees individually, eliciting comments/participation from all attendees, and comfort in diffusing/managing conflict.
3. **Technical**, with 4 items that focus on the facilitator’s grasp of important information, including knowledge of the CSC process and of the clinic’s standard health services package, to lead the discussion.

While facilitation quality is important throughout the CSC process, it is vital to the fourth step of the process: seeking shared solutions.

The facilitation checklist is intended to help staff focus on the importance of quality facilitation and enable supervisors to identify tangible areas for improvement. Either the health lead or the monitoring, evaluation, and learning (MEL) lead completes the checklist. Initially, there was some hesitation about the checklist; however, after ongoing use, it was seen less as a punitive tool and more as a valuable tool to prepare for facilitation and support ongoing facilitation improvement. Furthermore, the tool is often used to observe multiple facilitators who collaborate in facilitating the CSC process. Now, sites use the checklist as a self-reflection for facilitators to remind themselves ahead of time how to prepare for sessions and to review together after sessions what they think went well and where they can improve. For example, in one site in the Dominican Republic, the stakeholder meeting was delayed due to logistical challenges. This resulted in facilitators speeding the process, missing the opportunity to share important information pertinent to the CSC process. Several stakeholders left prior to concluding the session due to other commitments. Afterwards, the entire team, including the health supervisor and MEL lead, used the checklist to identify challenges and gaps. The checklist helped identify logistical issues that could be mitigated in the future to start on time. In addition, the checklist was used at the end of the day in preparation for the following day’s activities for immediate continuous improvement. At the organizational level, facilitation quality can be reviewed across sites to identify where refresher training or additional programmatic support are needed. Based on these data, we identified that additional support on facilitation training skills is a gap and needs continuous reinforcement, especially with staff turnover.

### Score Card

During Step 3, situation analysis, the indicator scoring takes place. Prior to indicator scoring, during the first cycle the team facilitates focus groups with each stakeholder group (providers, users, youth, and leaders) to understand the state of health services based on their perceptions, attitudes, experiences, and expectations. The staff and a representative from each group highlight key themes from the focus groups, grouped by 4 global indicators that are used across all sites: quality of care, availability of medical staff, availability of medicines and supplies, and community involvement/participation.

The stakeholder groups gather again the next day to learn about the global indicators, their definitions, and key themes from the focus groups, creating a common understanding of the indicators among all stakeholders. Each stakeholder group then assesses the situation of the clinic from their experience and based on the information provided and reaches consensus through small group dialogue on a score from 1–100 for each indicator, with higher scores indicating more positive experiences. In some cases, a program staff member helps facilitate the small group if no one from the group decides to facilitate. Each stakeholder group then shares their scores and experience with the entire committee. These data are then used to generate dialogue and build consensus across all stakeholder groups as they determine priorities and strategies for action. The cycle repeats every 6 months, including rescoring the indicators in Step 3.

Common understanding of indicators across stakeholder groups and consistent understanding across cycles was a challenge early on. This led to enhancing the definition of the global indicators in the implementation manual and ensuring clarity prior to scoring during the facilitation. The implementation manual defines the indicators as follows:

- **Quality of care** from the user’s point of view refers to appropriate care, respect, dignity, and clarity of communication. It also means that a provider speaks well and answers questions in a pleasant tone; that waiting time is reasonable and according to the country’s standard and adequate to user expectation; that privacy and confidentiality are respected; and that there is an easy way for users to present their complaints and recommendations.
- **Availability of medical staff** refers to the presence of medical staff in sufficient quantity and quality that guarantees care when users need it, without excessive delays.
- **Availability of medicines** from the user’s perspective will be determined by what is received in relation to what is prescribed and delivered. Examples of challenges identified through the focus group discussions are lack of medicines, use of the same medicines to treat different diseases, and dispensing medications that are close to their expiration date.
- **Community participation** is based on the Pan American Health Organization and World Health Organization concept that community participation is not only an end but also a means to improve health equity, efficiency, and quality of service, and to promote public health in general. For example, issues that have been identified during the focus groups are users do not participate and leave the center dirty, are late or absent for appointments, do not have appointments but still want to be seen, or community leaders seek care based on privilege.

These global indicators were identified as the most common indicators across multiple sites during the pilot phase and, thus, important areas to track progress. While all sites score the 4 global indicators, sites can also include additional relevant indicators that emerge out of focus group discussions during the situation analysis. For example, one site in the Dominican Republic identified insecurity around the health clinic as a challenge and barrier to accessing health care. By adding health clinic security as an indicator to monitor regularly, leaders kept the issue at the forefront of their minds, which led to security improvements.

**FIGURE 3:**
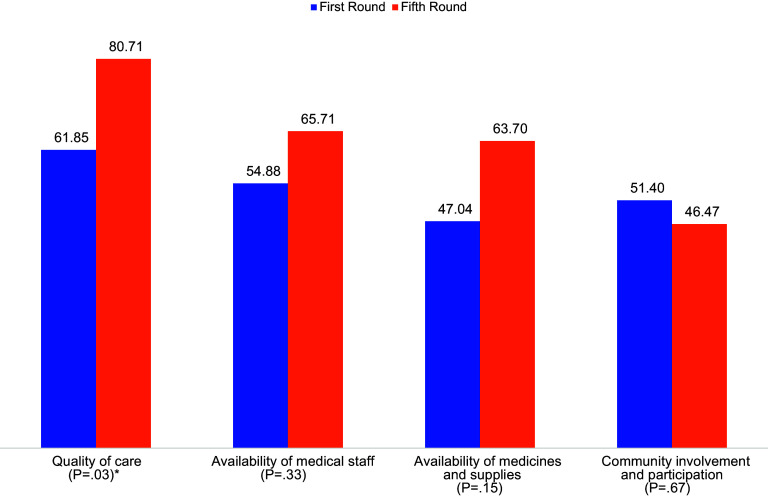
CSC Participants’ Mean Score (0–100) of Global Indicators, First Cycle to Most Recent Cycle, 7 Sites in the Dominican Republic, 2018 to 2023 Abbreviation: CSC, Community Score Card.

Figure 3 shows the change in the 4 global indicators from the first to the fifth cycle of scoring across 7 sites in the Dominican Republic. Improvements are seen in 3 of the 4 indicators: quality of care, availability of medical staff, and availability of medicines and supplies. Quality of care, in particular, was found to have statistically significant improvements between rounds (*P*=.03 from t-test), increasing from about 62% to 81%. Community participation as a global indicator looks low when aggregating across sites. While this indicator increased in most sites, one site initially scored very high on community participation and then dropped dramatically, contributing to the overall decrease in the aggregate. There was a gap in personnel at the site that created some challenges in consistent implementation and there were also a few people involved that were utilizing the CSC space to advance their own political career goals during an election cycle. Both situations created a reduction in community participation. Ensuring staff are adequately prepared to manage power dynamics when facilitating the CSC process is important as is working to maintain consistent staffing, especially during the early cycles.

### Action Plans

The Action Plan tab in the toolkit allows sites to summarize the activities prioritized by stakeholders to address areas of concern during each Seeking Solutions meeting (Step 4) and then to track progress on those actions from cycle to cycle. Actions are grouped under the indicator they are designed to address, so that participants consider what tangible actions they want to take toward improving each indicator and at the same time can point to tangible actions accomplished when indicators improve over time.

During the first Seeking Solutions meeting, the Action Plan is a planning tool. Over the next cycles, it also becomes a tracking tool. Before participants prioritize new or additional actions in the next Seeking Solutions meeting, they review progress on the actions planned during the previous round, marking each action as “completed” (green), “substantial progress” (yellow), “little progress” (orange), or “not started” (red). There is also a column to add notes about reasons for the status. The Action Plan provides sites with a visual, color-coded representation of what they have accomplished and where they have faced challenges. It also allows local staff the opportunity to share tangible accomplishments with government officials and other key stakeholders. At the organizational level, Action Plans provide documentation of the specific activities that sites are implementing and help identify any common barriers to progress across sites that can be addressed or supported by the organization.

There are several examples of concrete actions taken across the 10 sites because of the CSC process:

- **Availability of medicines and supplies:** Multiple sites have organized to ensure a consistent supply of medicines. One site has established a commission to oversee the management of medicines and track supplies. At another site, the committee advocated for medicines at the national level and secured a more consistent supply.
- **Cleanliness of facilities:** Several locations raised concern about the cleanliness of facilities through the CSC process. At one clinic, the committee organized several clean-up days for both the interior and exterior of the clinic. At another, the committee created a rotation schedule for cleaning the clinic. At a third clinic, the committee petitioned the city council for regular collection of garbage from the clinic.
- **Patient–provider relations:** Relationships between patients and providers was raised as another area of focus in multiple sites. At one site, the committee held an informational meeting for the community to discuss the services offered by the clinic and the relationship between patients and providers. At another, the committee held 2 meetings with providers to discuss concerns about patient–provider relationships. Finally, another committee secured a donation of benches for the waiting room, chairs for the doctors, and fans to use in the minor surgery room because the CSC process identified that these items would make the clinic environment more pleasant for both patients and providers.

Overall, the themes related to quality of care—that is, issues related to cleanliness within and around the clinic and treatment of patients by medical staff—appear in multiple sites, with action plans consistently showing green, suggesting that the issues were addressed successfully. Such quality-of-care issues are fairly easy to address and achieve as they are within the immediate control of the committee to influence. Other action items that are seen across multiple sites relate to availability of medicines and availability of health personnel. These action items require making requests to government officials and the providers of medicine, which typically require small incremental steps across multiple cycles to create change as they are more dependent on different levels of government to respond.

One challenge observed with the action plans is a lack of consistency with tracking ongoing actions that were not able to be completed from cycle to cycle. In cases where the action was deemed unfeasible or deprioritized, the reason for discontinuation was not always documented clearly. Also, additional training and reinforcement are required in the future to add adequate detail to document what happened to improve understanding for someone outside of the immediate committee.

### Empowerment Survey

The CSC intervention is designed to empower those most vulnerable and marginalized in the community to participate, speak up, and have their voices heard in collective action. Because empowerment is a central tenet of how social accountability interventions work to create change, we measure and track participants’ level of empowerment over time through a survey adapted from a tool developed and validated by CARE among women in Africa.^38^ The survey items measure participants’ comfort in speaking out at community meetings under various scenarios, their perceived treatment at community meetings, and their perceived ability to work together with different facets of the community to effect change,^38^ as well as participant characteristics such as age, gender, key stakeholder group, and whether they are a first-time participant. The survey is administered during every Seeking Solutions meeting (Step 4) to provide a measure of change between cycles.

Initially, we developed the survey as a self-administered instrument but faced some challenges due to low literacy levels among many participants. Several sites therefore shifted to an interview-administered format, increasing both the time needed to complete the surveys and the burden on staff who must administer them. Furthermore, while staff are trained to facilitate and not influence responses, there is potentially some response bias introduced.

**FIGURE 4:**
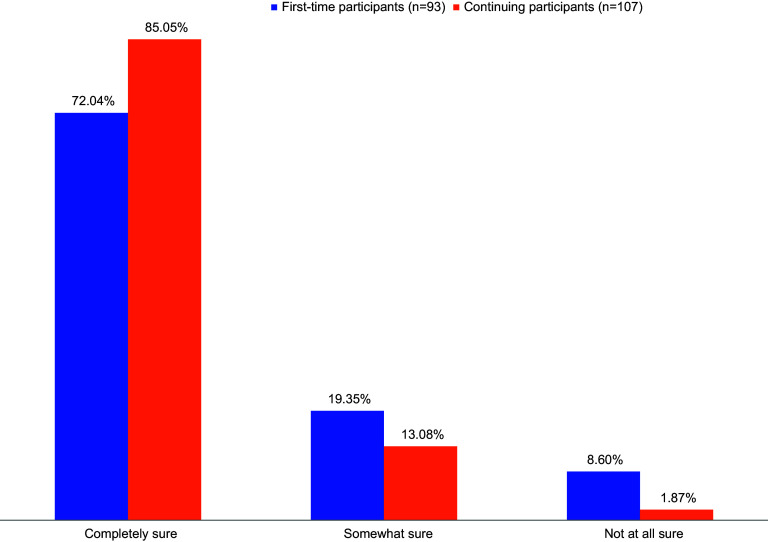
Confidence in Ability to Express Opinions at CSC Meetings Among Participants, 7 Sites in the Dominican Republic, 2022 to 2023 (N=200 Participants) Abbreviation: CSC, Community Score Card.

**FIGURE 5:**
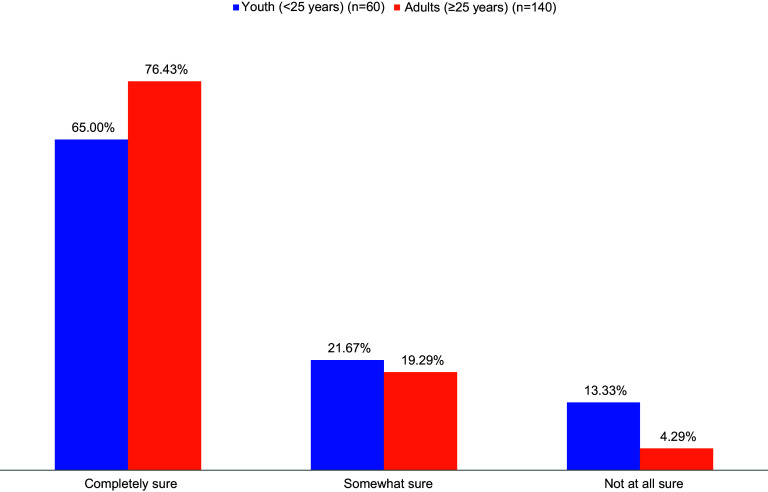
Confidence in Fair Treatment at CSC Meetings Among Participants, 7 Sites in the Dominican Republic, 2022 to 2023 (N=200 Participants) Abbreviation: CSC, Community Score Card.

In the Dominican Republic, analysis of empowerment surveys found that stakeholders who participated more than one time in the CSC process were significantly more confident in their ability to express their opinions than those participating for the first time (χ_2_^2^ = 6.8, *P* = .03) (Figure 4). Youth reported lower confidence than adults in being treated fairly during sessions (χ_2_^2^ = 5.8, *P* = .06) (Figure 5). This reiterates the need to engage youth in leadership and facilitation roles of the CSC process as the 3 sites in the Dominican Republic did.

Local staff used the data to identify how comfortable participants are speaking out at community meetings and their perceived ability to work together with different facets of the community to effect change. The Dominican Republic team also reflected on gaps between the survey responses and how people interacted during the scoring activity, using this information to inform adjustments needed. However, the team also faced challenges in analyzing the data due to some issues in the survey administration system and set up. These issues have since been resolved, and the team is interested in using the data more consistently in subsequent cycles to better understand ways in which the data can be used.

### Most Significant Change

Most Significant Change (MSC) is a qualitative data collection and analysis technique designed to elicit specific stories from the personal perspective of participants that epitomize how an intervention affected change.^39^ We use MSC every third cycle, or approximately every 18 months, to capture changes that may take more time to percolate. The original MSC methodology uses a vertical filtering process wherein each level within an organization selects the stories that they feel reflect the most significant change from the level below, until a single story is selected to represent the overall most significant change. We initially tried a horizontal filtering process: During the pilot and training meeting in the Dominican Republic, facilitators grouped the 17 participants into key stakeholder groups—providers, youth, community leaders, and service users. Each participant had the opportunity to write and share a story of significant change in their small group. The first layer of filtering took place within each stakeholder group. They discussed and selected a story from those shared in their group that best illustrated their perception of how the intervention created significant change. Then the stakeholder groups presented their stories to the overall group and collectively decided which story represented the most significant change, so that the final decision itself came from the participants themselves. This led to documented stories from each stakeholder group and 1 story of the overall MSC experience from the collective group.

#### MSC Challenges

Horizontal filtering with all stakeholders involved proved challenging, as individual participants and key stakeholder groups were wedded to their own story and did not want to claim any story as most significant. Instead of selecting a single story, they settled on a composite story that contained elements of all the proposed stories. While these composite stories did offer some value and insight, we decided to adjust the last step to reflect the MSC methodology more precisely. Each small group identified 1 story from the many that were expressed in their group to represent the story of the most significant change to them. Then local staff and members of the Solutions Committee considered the stories selected by each small group and, in turn, selected the 1 story they thought best represented the changes occurring in their site and shared it with the headquarters office to use as evidence of impact and examples to share with local stakeholders. Learnings from the experience in the Dominican Republic also led to improvements in the MSC documentation template to better capture all stories, not just the selected stories, and to ensure adequate detail was recorded to truly represent the MSC.

Efforts were made to train facilitators in advance and create an open environment prior to starting the MSC process. Each group had a staff member to support facilitation in order to provide an opportunity for each person to express their story and reduce bias of 1 person dominating the conversation. In some cases, each person in the group first wrote their own personal story, then shared them verbally after writing. This helped to reduce bias from listening to other stories. Other challenges we found were that participants expressed only positive stories and most stories were written from an observational perspective as opposed to a personal experience. In future efforts, we will probe participants to provide personal stories of change and clarify that there could be negative stories. As of this writing, MSC has only been implemented in 1 location, but plans remain to implement in remaining sites and to incorporate learnings from the initial round.

#### MSC Results

All stakeholder stories selected from youth, providers, and the community, challenges with cleanliness and hygiene of the clinic and poor treatment of patients by unqualified medical staff were mentioned as problems before the CSC initiatives, leading people to avoid seeking services at the clinic. The stories indicated that the CSC initiatives led to improvements in cleanliness, hygiene, and better treatment from providers, and they expressed a strong sense of pride that their efforts and collective action led to changes. During the dialogue after sharing each story, there was agreement that the community felt empowered to make changes and to have ownership of the results.

For example, the youth MSC story expressed that when people tried to access services, medical staff responded with scolding and bad temper. Furthermore, the center “was perceived as dirty.” As a result, users sought medical care at a secondary- or tertiary-level center to avoid insults, dirt, and the bad temper of the staff. The following is an excerpt from the youth MSC story:

*You go to seek health, not to get sick. We implemented activities to develop empathy between the parties [providers and health service users]. Motivational posters were created inviting service providers to put themselves in the users’ shoes and vice versa. Support was provided to the [clinic] to maintain cleanliness in the center. Now we have cleaner areas and dignified treatment everywhere: the changes are visible, and the community expresses it. Good treatment and cleanliness are making people feel more confident in accessing health services; they express that today “it is a pleasure to go to the clinic.”*

This is important because the community is regaining confidence in medical care, attending scheduled consultations, and feeling comfortable being treated by the staff.

The providers’ MSC story explains that before the CSC the community was not involved in health services. The management of supplies and the cleanliness of the center, both inside and outside, were not a concern for the community. Abandoned vehicles, tires, and deteriorated areas around the health center were breeding grounds for mosquitoes, emitted bad odors, and showed a neglected state of hygiene, which affected people’s access to medical care.

With the CSC, the community began to participate in a collaborative effort to improve healthservices. They communicated with the mayor to request the removal of sources of contamination (abandoned vehicles) and the beautification of the area, and action was taken.

*The health provider expressed [that] the clinic now has a social fabric willing to improve the user experience of health services. Continuous feedback is practiced; health providers now see community members and service users as allies.*

The community-selected story expressed similar challenges of cleanliness and quality health providers and a change to coordinated and aligned efforts to create change. In addition, they expressed that there were fragmented visions and efforts among community organizations and there was mistreatment, lack of medications, and the need for competent personnel to perform the work of a doctor. The person acting as a doctor was not qualified, putting the health of the service users at risk. Through CSC, the different community groups united and built a common agenda. They implemented activities to improve good treatment, took advantage of links with public institutions to remove abandoned vehicles that produced mosquitoes, and carried out fumigation campaigns in the community. They beautified the surroundings and became the main promoter of the health services offered by the clinic in the community. They even advocated to the government to assign qualified personnel with the appropriate competencies and technical accreditation.

*The collective work has restored hope and belief in local health services; people who stopped attending the [clinic] due to [poor] hygiene and mistreatment have returned and enthusiastically indicate that the change is real.*

The MSC process has proven to be powerful for each stakeholder group. The participants reflected on the progress made and saw the impact coming from each stakeholder group. The group reflection of the stories highlighted the empowerment and confidence that the stakeholders felt in being able to identify challenges, align goals, and work together to achieve changes to improve the health services. After the process, the stakeholders were inspired and proud of what they accomplished, and it generated more engagement. The Dominican Republic team reported that those who participated in the MSC were more regular participants in activities afterwards.

MSC provided local staff with tremendous inspiration seeing the impact of the CSC from the participants’ perspectives. The local staff have used the stories for advocacy at higher levels of the health system for the transformation of health services and to demonstrate to partners and strengthen national-level partnership support for CSC. In one example, MSC stories were shared at a regional pre-symposium meeting for Latin American and the Caribbean in advance of the Health Systems Global Symposium, highlighting the involvement of the community and youth in the strengthening of the health system.^40^ The stories have also been shared with other countries launching CSC to understand what is possible and to gain buy-in for the process.

## LESSONS LEARNED

### Toolkit Learnings

Three key lessons arose from the development and implementation of the CSC Monitoring and Evaluation Toolkit. First, a *comprehensive toolkit* is helpful to have all data in one place and to document evidence from multiple sources together. This allows for triangulation of data and a common data set that all stakeholders can explore, analyze, and use. The data captured through the toolkit demonstrated evidence of community empowerment based on the variety of data sources.

Second, there is *value in a global standardized toolkit* that allows for analysis across sites. This toolkit provides the ability to analyze data across sites and globally while also allowing for local contextualization in the approach and elements. For example, the Dominican Republic team can aggregate data across 10 sites to demonstrate the advancements they achieved to the National Health Service and Ministry of Health to bolster support and buy-in for the process while using local indicators to speak to the community and local leadership about a particular site. We found the value of global indicators outweighed the limitations.

Finally, *ensuring data are actionable locally* is important to gathering the complete, accurate data necessary for continuous quality improvement. Developing the toolkit collaboratively with all stakeholders and adapting it over time helped ensure it was useful and intuitive. Ongoing training, visualization of data, and dedicated resources to analyze the data and findings are necessary. Further exploration of ways to automate data collection and populate visualizations more frequently is recommended for the future to improve data consistency and use and to make data management easier at the community level.

### Process Learnings

The toolkit development enhanced the ability of the Dominican Republic team to collect data to understand areas for improvement in the CSC process, specifically youth involvement and the importance of achieving early, small successes by the committee.

Involving youth as *participants* in the traditional CSC process was not enough. Instead, the Dominican Republic team found that supporting youth-*led* mechanisms helped to ensure that youth voices were heard and elevated. The youth-led approach involved preparation and awareness-building for youth on their rights, the health system, and advocacy. By including youth in the design and facilitation of the CSC between providers and young people, their unique perspectives were heard and prioritized into actions. Youth leadership presented challenges and required creativity and flexibility to work within the schedules and priorities of young people. This can mean shorter-term commitments and plans due to studies or work. The Dominican Republic team’s learning in this area aligns with findings presented in a youth landscape analysis that recommended investing in youth-led organizations and movements, strengthening adult capacity to partner meaningfully with youth, and addressing social norms that limit the value and power of young people.^20^ In future evaluations, we plan to explore how to connect youth-led social accountability to positive youth development outcomes.

To achieve higher-level health systems changes, such as increased personnel or medicines and supplies to meet the demands of the community, it is important to begin with small locally led change. In the Dominican Republic actions such as community clean-ups of the health facility, awareness campaigns, and relational improvements between health care providers and community members were changes within the control of those participating in the CSC process. These improvements led to increased trust among stakeholders and collective efficacy that bigger change is possible. Through these early wins, the process and committees gained credibility and motivation to take on higher-level actions collectively. Through this process, local health care providers and community members worked together to advocate for more sustainable change, and the past improvements supported further vertical integration and support from higher-level decision makers.

### Institutionalization, Sustainability, and Scalability

Our experience in the Dominican Republic has also helped inform considerations around institutionalization, sustainability, and scalability. In terms of sustainability, the CSC is facilitated through a stakeholder representative process. The stakeholder-appointed committee determine their own governance, including duration of service on the committee and roles and responsibilities, and leadership is expected to evolve over time. Through the CSC process, local capacities are strengthened among stakeholders, ensuring it is a community-led process of cooperative engagement of both health care providers and users in ensuring accountabilities for improved quality of health services. Trust and cohesion of the committee is evidenced in the Dominican Republic and this helps seed into the sustainability and ability to continue in other sites.

In terms of institutionalization, the involvement of government leaders and government-run health clinic staff has provided opportunities for institutionalizing the committees to respond to the government’s desire for citizen engagement in strengthening health clinics. Creating a collaborative space where both health care providers and the community feel empowered, engaged, and supported by each other to drive change helps foster commitment to the process.

Based on CARE Malawi’s sustainability study,^41^ we know that interface meetings can be challenging to maintain. We continue to strengthen the capacity of the CSC committee over time and to use the toolkit data to demonstrate to local government and leadership the benefits of the CSC to build commitment for resource sharing and funding. As more sites complete 6 or more rounds of the CSC, sustainability and institutionalization will continue to be explored further.

In terms of scalability, other health care clinics in the Dominican Republic are now using the same approach in areas beyond our catchment areas of operation. We have shared best practices in feedback mechanisms that have supported more community inclusion and equitable service provision. Furthermore, we have used lessons from our experience in the Dominican Republic to scale the CSC to other country agencies and health facilities across 8 countries in which we operate. The learnings from our CSC experience in the Dominican Republic led to the creation of the CSC implementation manual, and the Dominican Republic team were leaders in training other country staff to implement CSC. Furthermore, the learnings about youth involvement in the Dominican Republic informed ways in which other countries engage youth. Examples of action plan accomplishments in strengthening the clinics provided inspiration and motivation for other teams to buy into the process and have confidence to launch CSC in their countries.

Finally, reflecting both the sustainability and scalability of the CSC process, representatives from the government of the Dominican Republic expressed a desire to expand the CSC to more clinics across the country where our organization is not working. The government has invited our staff to train facilitators at other clinics and share their lessons learned about the process. This scale up was due in part to word-of-mouth promotion from both community members and providers involved in CSC and demand from other areas to have something similar in their community. The government’s interest in expanding the CSC process reflects how the process can help meet the government’s goal of more community engagement and feedback to improve quality of care and satisfaction.^32^

## CONCLUSION

Overall, the comprehensive CSC monitoring and evaluation toolkit provided the ability to document case study data on the social accountability approach being used in the Dominican Republic, including capturing evidence of the change. Continuous quality improvement in the CSC process led to promising results, as evidenced by the score card data and Most Significant Change stories, showing improvement in the 4 defined global health service indicators of quality of care, availability of medical staff, availability of medicines, and ongoing community participation including youth. Studies show that improving health service users’ perception of the services leads to increased uptake of the services and improved health outcomes over time.^26^ In addition, the stakeholders demonstrated increased morale for engagement and community empowerment to drive change, which contributes to strengthening local health care services. This was apparent in the attendance data, improvements in the score card, and the Most Significant Change stories. This reinforces and supports the Dominican government initiatives to improve health care delivery through increased community participation and accountability in health clinics.^30^ Overall, the comprehensive CSC monitoring and evaluation toolkit supports efforts for continuous quality improvement while producing evidence locally, nationally, and internationally for health systems strengthening and demonstrating the effectiveness of the CSC approach.

## Notes

**Peer Reviewed**

**First Published Online:** March 13, 2026.
